# Comparative Study of the Efficacy and Tolerability of Palmex^®^ (*Roystonea* *regia* Lipid Extract), Saw Palmetto, Finasteride and Tamsulosin in Patients with Benign Prostatic Hyperplasia

**DOI:** 10.5152/tud.2025.24067

**Published:** 2025-03-06

**Authors:** Raúl Guzmán Mederos, Mónica Reyes Bello, Julio César Fernández Travieso, Manuel Pedroso Gastón, Sigfredo Calzadilla Feijoo, Miriam Hernández Rech, Maria de los Angeles Viamontes Lu, Yenney Reyes Nuñez, Jilma Mena Figueroa, Zunilda Rodríguez Suárez, Lai López Rodríguez, Niurka Basulto Turran, Yolanda Cruz Gómez, Meilis Mesa Angarica, Sarahí Mendoza Castaño, Maytee Robaina García, Gladys Jiménez Rivero

**Affiliations:** 1 Urology Service, ¨Salvador Allende¨ Hospital, Havana, Cuba; 2 Urology Service, Medical Surgical Research Centre, Havana, Cuba; 3 Clinical Trial Unit, National Centre for Scientific Research, Havana, Cuba; 4 Clinical Laboratory, ¨Salvador Allende¨ Hospital, Havana, Cuba; 5 Clinical Laboratory, Medical Surgical Research Centre, Havana, Cuba; 6 Research Department, National Centre for Scientific Research, Havana, Cuba; 7 Statistical Analysis, National Clinical Trials Coordinator Centre, Havana, Cuba; 8 Date Management, National Clinical Trials Coordinator Centre, Havana, Cuba

**Keywords:** Benign prostatic hyperplasia, finasteride, palmex, saw palmetto, tamsulosin

## Abstract

**Objective:**

Evaluate and compare the efficacy and tolerability of the treatment with Palmex^®^, saw palmetto, finasteride, or tamsulosin administered for 6 months to patients with benign prostatic hyperplasia (BPH).

**Mthods:**

This multicenter, open, prospective, comparative study was conducted in men ≥40 years with mild and moderate BPH according to the International Prostate Symptoms Score (IPSS) (≥1, <19). The study included 200 patients (50 per group) who were randomly allocated to receive Palmex^®^ (320 mg/d), saw palmetto (320 mg/d), finasteride (5 mg/d), or tamsulosin (0.4 mg/d). The primary study outcome was the improvement of the maximum urinary flow (Qmax), while significant decreases in IPSS values, prostate size, and residual post-voiding volume were secondary efficacy variables. Statistical analysis was performed according to Intention to treat.

**Results:**

The demographic baseline characteristics of all the groups were similar. All groups exhibited a significant mean increase in Qmax from baseline to week 24, being 3.8 mL/s (27.7%), 3.6 mL/s (23.1%), 4.2 mL/s (28.6%), and 4.1 mL/s (26.3%) on Palmex^®^, saw palmetto, finasteride, and tamsulosin, respectively. Likewise, all the treatments significantly reduced the IPSS scores by 74.2% (Palmex^®^), 74.6% (saw palmetto), 60.3% (finasteride), and 74.2% (tamsulosin), also decreasing the prostate size and the residual post-voiding volume. No significant differences among the groups were found regarding any efficacy variable. The treatments were well tolerated.

**Conclusion:**

Palmex^®^ for 6 months demonstrated to have a comparable efficacy to saw palmetto, finasteride, and tamsulosin in patients with mild and moderate BPH, being safe and well tolerated.

**Main Points**

- This study reports for first time, the effects of Palmex® 320 mg/d given for 6 months on the urinary Qmax an objective flowmetric variable selected as the primary study outcome.
- Palmex® shows that its efficacy on the primary (Qmax) and secondary efficacy outcomes (IPSS, prostate size, residual post voiding volume) was comparable to that of saw palmetto, terazosin and tamsulosin in patients with mild and moderate BPH.
- Treatment with Palmex® was safe and well tolerated.

## Introduction

Benign prostatic hyperplasia (BPH) is the most common urological disease, which mainly affects men ≥40 years, and whose frequency increases with age. Benign prostatic hyperplasia consists of the benign but excessive and uncontrolled growth of the prostate, including both stromal and glandular elements, which can cause obstruction and promote the development of lower urinary tract symptoms (LUTS), such as decreased urine volume and urination pressure, urinary retention, and nicturia. These symptoms commonly affect the quality of life of affected men.^1^

While mild BPH can be managed by the “watchful waiting” approach, when drug treatment is required, moderate and severe BPH often require monotherapy or combination therapy. Most patients are managed with selective α1-adrenoceptors blockers, while those with larger prostate volumes may benefit from a 5-α-reductase inhibitor, usually in combination with an α-blocker, this combination being recommended mainly for severe or refractory BPH.^2^

On their side, phytotherapeutic alternatives, albeit not approved in urological guidelines to manage BPH/LUTS, have been used in patients with BPH. Among these, saw palmetto (*Serenoa repens B*) (Arecaceae) extracts are the most documented,^3^ although its efficacy has been controversial with negative studies published.^4^

The lipid extract from the fruits of the Cuban royal palm (*Roystonea regia*) (named D-004 by former research code) has been effective in experimental models of prostate hyperplasia,^5^^-^^7^ with an efficacy similar to saw palmetto based on a mechanism that involves two basic components: a) inhibition of prostatic 5α-reductase,^8^ a fact that supports its effects on the mechanical component of the disease; and b) antagonism of responses mediated by ADR-α1, demonstrated “in vitro” and “in vivo”,^7^ which supports its experimental efficacy on the dynamic component of the disease. In addition, antioxidant effects of the extract on rat prostatic tissue have been shown.^9^

In turn, experimental toxicology demonstrated that oral administration of the extract was safe when given for short and long terms to rodents and dogs, and that no genotoxicity, reproductive toxicity, or long-term carcinogenicity effects were found.^10^,^11^

Clinical studies have demonstrated the efficacy and safety of Palmex in short-, medium-, and long-term trials in patients with BPH.^12^^-17^

Despite these grounds, no previous study has investigated the efficacy of Palmex® using as primary study outcome an objective urinary measure like maximum urinary flow (Qmax). Considering these issues, this study compared the effects of Palmex^®^, saw palmetto, finasteride and tamsulosin, administered for 6 months, on Qmax values in patients with BPH, including LUTS and other variables as secondary outcomes; also assessing their safety and tolerability.

## Material and Methods

### Study Design

This multicenter, open, randomized, prospective, comparative study was conducted according to the Declaration of Helsinki.^18^ Study protocol and procedures were approved by the Ethics Committee in Clinical Research of both Hospitals, Salvador Allende Hospital and Medical Surgical Research Centre (Approval No: IRB220321 and IRB170621 Date: 03/22/2021 and 06/17/2021). This study was included in the Cuban Public Registry of Clinical Trials (RPCEC00000373).

All participants (men previously diagnosed with mild or moderate BPH) provided written informed consent at enrollment. Their clinical history, responses to the International Prostate Symptoms Score (IPSS) questionnaire, and physical examination were recorded for screening their eligibility for randomization (visit 1).

Eligible patients were randomly allocated to receive Palmex^®^ (320 mg/d), saw palmetto (320 mg/d), finasteride (5 mg/d), or tamsulosin (0.4 mg/d) once daily for 6 months, being advised to continue their usual dietary habits. Further visits were done after completing 2, 4, and 6 months on therapy (visits 3-5). Subjects underwent physical examination and IPSS interview at each visit. Treatment compliance and adverse events (AE) were monitored from visits - 5. Laboratory tests and ultrasound evaluations were conducted at baseline and at study completion.

### Study Participants

Men aged ≥40 years and previously diagnosed with BPH were enrolled in the trial, being eligible for randomization if they corresponded to cases with mild or moderate BPH (IPSS ≥ 1, <19), and had enlarged prostates confirmed by both digital examination and ultrasound.

Exclusion criteria included any major prostate conditions other than BPH, prior urogenital surgery, urinary retention, neurogenic bladder, prostate-specific antigen (PSA) values >4.0 ng/dL, and/or creatinine levels >177 μmol/L. Additional exclusion criteria were blood pressure values >180/110 mm Hg, mental health problems that limited reliable answers to the IPSS questionnaire, diagnosed neoplasias, and/or serious vascular events (acute coronary syndromes, stroke, transient ischemic attacks, and/or major surgery) during the prior 6 months.

Apart from study drugs, consumption of other medications and/or supplements with known effects on BPH/LUTS and/or on urinary parameters was not allowed during the trial. Patients who were taking some of them were eligible for randomization only if they stopped the consumption for at least 6 months prior to the trial.

Causes of premature withdrawals included suffering any adverse event justifying such a decision, unwillingness to continue the study, and major violations (failure in taking study treatments for ≥15 days and/or taking supplements or medicines with known effects on BPH/LUTS).

### Treatments

Palmex^®^ and saw palmetto were administered as 320 mg opaque softgel capsules, finasteride as 5 mg tablets, and tamsulosin as 0.4 mg hard capsules.

To ensure equity in treatment allocation, identical packages of each treatment were given to the patients according to their serial progressive inclusion in the trial. Randomization was computer-generated using balanced blocks and an allocation ratio of 1:1, without stratification.

Patients were advised to take the treatments once a day after dinner for 6 months and to bring unused capsules or tablets to each visit. Treatment compliance, estimated by capsule or tablet counts at each visit and by patient interviews, was considered good and very good if they consumed ≥80% or ≥90% of the units scheduled from the previous visit, respectively.

### Efficacy Outcomes

The primary efficacy outcome was to determine whether the daily use of the treatments for 6 months improved the values of Qmax, an objective measure of urinary obstruction, as compared to baseline. Secondary efficacy outcomes included the reduction of LUTS as measured by the IPSS, the decrease of residual post-voiding volume measured by ultrasonography, and the reduction of prostate size.

Treatments were considered effective if they produced a significant increase in Qmax, and/or a significant reduction in IPSS versus baseline values. A reduction in the residual post-voiding volume and prostate size were additional elements for the overall evaluation of efficacy.

The efficacy of Palmex® 320 mg/d was considered comparable to that of classical prescription drugs (tamsulosin and finasteride) if the final values and percent changes of Qmax and IPSS among these groups were statistically similar. Saw palmetto was used as the phytotherapeutic nearest to Palmex^®^ in composition, dose, and formulation.

Also, the responder rate at study completion based on the improvement from baseline in Qmax and IPSS was analyzed, defining a responder as a subject with a Qmax increase ≥20% and an IPSS reduction ≥40% from baseline without requiring additional therapy.

### Safety and Tolerability

Data from physical examinations, laboratory safety indicators, and AE were analyzed. Safety indicators included physical (body weight, pulse rate, blood pressure), hematological (hemoglobin, hematocrit, platelets, blood red and white cell counts), and blood indicators (prostate-specific antigen (PSA) levels, alanine aminotransferase (ALT), aspartate aminotransferase (AST), glucose, and creatinine).

All undesirable events that newly appeared in a patient during the trial, regardless of the cause, were considered AEs. In accordance with their intensity, AEs were classified as mild, moderate, or serious. Mild AEs should not require the suspension of study drugs and/or specific treatment of the AE, moderate AEs should require stopping therapy and/or specific treatment of the AE, while serious AEs should lead to hospitalization and/or deaths.^19^

Drug-related causality was assessed according to the WHO-UMC system for standardized case causality assessment.^20^ In such regard, AEs were classified into one of the following causality categories: certain, probable/likely, possible, unlikely, conditional/unclassified, or unassessed/unclassifiable.

### Laboratory Variables

Blood venous samples were drawn after an overnight fast of 8-10 hours. Hematological indicators were automatically determined using a hematological complex equipment. Blood biochemistry indicators were assessed using reagent kits (Roche, Switzerland) in a biochemical auto-analyzer (Tokyo, Japan). Prostate-specific antigen levels were determined by immunoenzymatic assay (UMELISA PSA kit, Immunoassay Center, Havana, Cuba).

### Statistical Analysis

Data analysis was conducted according to intention-to-treat principles, including all randomized patients regardless of compliance with the scheduled treatments, and data imputation for missed values was done using the carryover method.

Continuous variables were compared by using the *t*-test for paired samples (within-group comparisons) and ANOVA (between-group comparisons). Categorical variables were compared with the chi-square test.

All the statistical tests were 2-tailed and the significance level, established a priori for all the statistical tests, was *α* = 0.05; that is, for all *P* < .05 it was considered significant.

Data management and statistical analysis were carried out in the Department of Data Management and Processing of the National Coordinating Centre for Clinical Trials (CENCEC, by Spanish acronym).

## Results

### Baseline Characteristics of Study Patients

Of 233 enrolled patients, 200 were eligible for randomization. The causes of non-inclusion due to exclusion criteria were PSA >4 ng/mL (18 patients), prostate adenocarcinoma (1 patient), low hemoglobin due to digestive bleeding from an unknown cause (1 patient), high creatinine values (1 patient), and failure to carry out the indicated tests to be included (8 patients). Four patients could not be included because the sample size predefined in the protocol was completed.

Six patients (3%) withdrew from the study. Three withdrawals were due to AE (complete urine retention) (1 from each group, except for the finasteride one), while another 3 were due to unwillingness to continue by traveling abroad (2 from the finasteride and 1 from the tamsulosin-treated groups, respectively).

Table 1 shows the demographic characteristics of study population at baseline, all men with BPH (average age: 64 years old). Mean age, body mass index, Qmax, and IPSS values were well balanced in all the groups.

Compliance with study drugs was excellent. Except for the 6 withdrawals, the other randomized patients consumed all the scheduled treatments for each stage, as confirmed by remaining unit counts and patient interviews.

### Effects on the Primary Study Outcome

All groups exhibited a significant mean increase in Qmax from baseline to month 6, being 3.8 mL/s (27.7%), 3.6 mL/s (23.1%), 4.2 mL/s (28.6%), and 4.1 mL/s (26.3%) on Palmex^®^, saw palmetto, finasteride, and tamsulosin, respectively (Table 2).

Of the randomized patients, 26/50 (52.0%), 25/50 (50.0%), 23/50 (46.0%), and 27/50(54.0%) on Palmex^®^, saw palmetto, finasteride, and tamsulosin, respectively, exhibited a Qmax increase ≥20% at study completion, with the rate of responders being statistically similar in all the groups.

In addition, mean flow significantly increased in all groups by 27.5% (Palmex^®^), 23.1% (saw palmetto), 27% (finasteride), and 25.6% (tamsulosin), and voiding time decreased significantly by all the treatments, by 14.6% (Palmex^®^), 19.9% (saw palmetto), 23.5% (finasteride), and 20.6% (tamsulosin), respectively (Table 2).

### Effects on Secondary Outcomes

In turn, after completing 2 months of therapy, IPSS was significantly reduced versus baseline by 58.5%, 56.2%, 49.9%, and 61.9% with Palmex^®^, saw palmetto, finasteride, and tamsulosin, respectively (*P* < .001) (Table 3). This effect did not wear off; it was even more pronounced over time. At the end of the study, IPSS values were significantly reduced by 74.2% (Palmex^®^), 74.6% (saw palmetto), 60.3% (finasteride), and 74.2% (tamsulosin) as compared to baseline.

Palmex^®^, saw palmetto and finasteride treatments significantly reduced the mean prostate size vs. baseline. The post-void residual volume was modestly reduced in all the groups, but such a decrease reached statistical significance only in the Palmex^®^ group (Table 4).

### Safety and Tolerability

All treatments were safe and well tolerated. There were no significant changes in physical or lab safety indicators, and individual values remained within the normal range for each variable (values not shown for simplicity).

Eleven patients (5.5%) reported 16 AE during the study (Table 5). Three of these corresponded to withdrawals due to urinary retention referred to above. These AE were classified as moderate, managed with antibiotics and bladder catheterization, and then resolved. Also, 2 patients treated with saw palmetto, 2 with finasteride, and 4 with tamsulosin reported other AE, all classified as mild, some being sexual-related AE. All the AE reported during the trial were further classified as definitely treatment-related.

## Discussion

This study reports for the first time, the effects of Palmex^®^ 320 mg/d given for 6 months on the urinary Qmax, an objective flowmetric variable selected as the primary study outcome, and shows that its efficacy on the primary (Qmax) and secondary efficacy outcomes (IPSS, prostate size, residual post-voiding volume) was comparable to that of saw palmetto, terazosin, and tamsulosin in patients with mild and moderate BPH.

Despite these encouraging results, this study has 2 important design limitations: it was open and uncontrolled without including a placebo or plain control group. Thus, all comparisons were conducted versus baseline, making it impossible to determine if the subjective impression of all patients treated could influence both patients and evaluators, particularly regarding on IPSS results.

Study patients were included according to their IPSS scores (≥1, <19), representative of men with mild-moderate BPH/LUTS, although patients with moderate BPH/LUTS were predominant (164/200, 82%).

Main demographic characteristics, including average age (64 years old), concomitant diseases and risk factors, are consistent with data reported for patients with BPH/LUTS.^21^ Study participants had some concomitant diseases and risk factors, such as hypertension, smoking, and diabetes, among others, all of which harmfully contribute to the progression of the symptoms. The consumption of concomitant therapy in the study population was high (68.5%) and matches well with the personal history of the patients, so that antihypertensive drugs, oral hypoglycemic drugs, and antiplatelet agents were consumed by more than 10% of the randomized patients.

No significant baseline differences among the groups were seen, though the tamsulosin group apparently included a non-significant frequency of cases with moderately higher BPH/LUTS slightly higher than the others. This difference involved baseline IPSS scores that were somewhat higher, albeit not significantly, than the other groups.

Since baseline characteristics were statistically similar in all groups, they were homogeneous enough to consider the study results attributable to the treatment and not to disparities in the baseline condition of the study groups.

The frequency of study withdrawals (6/200 withdrawals, 3%) was low compared with dropout rates in reported studies;^22^ which indicates a good study conduction with minimal missed data.

Only 3 withdrawals were AE-related, 1 in each group, all due to urinary retention, except in the finasteride group. This result, although unexpected since finasteride tends to show higher AE-related dropouts in trials than tamsulosin,^23^ had not clinical relevance since there were no differences among the treated groups.

After 24 weeks of treatment, there were increases of 3.8 mL/s, 3.6 mL/s, 4.2 mL/s and 4.1 mL/s in Qmax in the patients treated with Palmex^®^, saw palmetto, finasteride and tamsulosin, respectively. The minimal clinically important difference for BPH/LUTS interventions has been proposed to be expressed as unit changes, being +2 mL/s for Qmax, so that all changes seen here can be considered clinically important. Also, these changes produced by saw palmetto, finasteride and tamsulosin generally agree with those reported in previous trials.^24^,^25^ Consistently, mean flow in all groups was significantly increased, and voiding time was reduced at study completion.

Likewise, IPSS total scores were significantly and markedly reduced (>50%) in all study groups, with decreases being of 74.2% (Palmex^®^), 74.6% (saw palmetto), 60.3% (finasteride) and 74.2% (tamsulosin), which were also were consistent with those reported by other authors.^24,25^ In addition, the standard deviation of IPSS values was ≤6.0, the cut-off assumed for their validity, indicating that the application of the scoring system was adequate.

The effects on IPSS values of Palmex^®^ and the other treatments were seen as soon as 2 months after therapy (first interim check-up) when all groups achieved reductions >40%, the cut-off considered for a MCIM to alleviate LUTS in patients with mild and moderate BPH and LUTS.^26^ This marked effect was enhanced over the treatment duration in all the groups. However, comparisons among groups did not show significant differences at the end of the treatment.

The frequency of patients achieving an IPSS decreases ≥40% was similar in the Palmex^®^ (46/50, 92%), saw palmetto (48/50, 96%), finasteride (45/50, 96%), and tamsulosin (48/50, 96%) groups, without significant differences among the groups. Similarly, the frequency of responders assessed according to the percentage of cases with net decreases in the IPSS score ≥3 points^27^ showed consistent results, since 44/50 (88%), 47/50 (94%), 42/50 (84%), and 48/50 (96%) of patients treated with Palmex^®^, saw palmetto, finasteride, and tamsulosin, respectively, achieved such reductions.

Despite results on saw palmetto for LUTS have been controversial, with some showing negative data and defending the concept that this treatment provides little to no benefits for men with LUTS due to BPH,^6^ other studies support positive results,^3^ which are consistent with those found in the present trial.

On the other hand, Palmex^®^, saw palmetto, and finasteride treatments were also effective for significantly reducing prostate size, a result which matches some previous ones. Curiously, the residual post-voiding volume was significantly reduced with Palmex^®^ only, an unexpected result considering the similarity of the treatments on all other variables. So, we do not consider this fact as an advantage of Palmex^®^ over the other treatments, but a random result without clear explanation.

The correspondence of the Qmax and IPSS changes with those reported for the reference treatments by independent studies,^24^,^25^ indirectly supports the study conduction and the effects of Palmex^®^ described here, although it doesn’t cancel the limitation of the open-label, uncontrolled design of the study to convincingly prove the therapeutic equivalence of Palmex^®^ with the other treatments.

Nevertheless, at least it provides some clue to accept this fact as preliminary evidence to be confirmed in further randomized, double-blind, head-to-head paired comparative studies with finasteride and tamsulosin.

Treatment with Palmex^®^ was safe and well tolerated, as expected, being consistent with previous data.^12^^-^^17^

Overall, all treatments can be considered well-tolerated, albeit an apparently higher, but not significant, report of sexually related AE was seen in the tamsulosin group, consistent with its AE profile.^28^,^29^

Research and clinical experience have reported adverse effects of 5-alpha-reductase inhibitors (5-ARIs) like finasteride and alpha-blockers like tamsulosin. These effects are primarily sexual and include erectile dysfunction, decreased libido, as well as gynecomastia^29^,^30^ for 5-ARI and postural hypotension, dizziness, asthenia, abnormal ejaculation, and intraoperative floppy iris syndrome in the case of alpha-blockers.^28^^,29^

However, no unexpected side effects were reported by study patients treated with finasteride. On the contrary, the frequency of patients reporting AE was significantly higher in the tamsulosin group than in the Palmex^®^ and finasteride groups, which do not match with comparisons between tamsulosin and finasteride,^29^ and seems to be related to the fact that the number of AEs related with sexual behavior, mainly ejaculatory disorders, was also significantly higher in this group.

In this regard, a recent systematic review and meta-analysis of the effects of alpha-adrenoceptor antagonists on sexual function reported that the odds for ejaculation disorders are greater in patients treated with selective or non-selective alpha-blockers than in matched placebo. Ejaculatory disorders associated with the administration of alpha-1 blockers are attributed to bladder neck relaxation, in turn causing retrograde ejaculation. Moreover, the odds for abnormal ejaculation are greater upon administration of the uroselective alpha-blockers tamsulosin and silodosin, consistent with a peripheral effect on the vas deferens and/or seminal vesicles and a central effect in the coordination of ejaculation.^28^

Finally, we must highlight the advantage, according to the results obtained in this study, of being able to have a therapeutic option (Palmex^®^) on the market with a similar efficacy to the most used medications for the treatment of BPH, but with a lower cost and financial burden for the patient.

Palmex^®^ for 6 months demonstrated to have a comparable efficacy to saw palmetto, finasteride, and tamsulosin in patients with mild and moderate BPH, being safe and well tolerated.

## Figures and Tables

**Table 1.** Main Baseline Characteristics of the Study

	Palmex (n = 50)		Saw Palmetto (n = 50)		Finasteride (n = 50)		Tamsulosin (n = 50)	
Age (years) (*X* ± SD)	65 ± 9		64 ± 9		63 ± 10		64 ± 8	
Body mass index (kg/m^2^) (*X* ± SD)	26.3 ± 4.3		26.2 ± 2.8		27.6 ± 5.9		27.8 ± 6.2	
Qmax	13.7 ± 9.8		15.6 ± 7.6		14.7 ± 9.6		15.6 ± 9.1	
IPSS score	10.8 ± 4.6		10.9 ± 4.5		10.0 ± 4.5		11.7 ± 4.5	
	3**n**	3**%**	3**n**	3**%**	3**n**	3**%**	3**n**	3**%**
Mild BPH/LUTS (IPSS ≥ 1, <7)	11	22.0	8	16.0	13	26.0	8	16.0
Moderate BPH/LUTS (IPSS ≥ 7, <19)	39	78.0	42	84.0	37	74.0	42	84.0
Personal history								
Hypertension	28	56.0	28	56.0	30	60.0	26	52.0
Overweight (kg/m^2^ ≥ 25, <30)	21	42.0	31	62.0	24	48.0	22	44.0
Obesity (kg/m^2^ ≥ 30)	9	18.0	4	8.0	13	26.0	12	24.0
Smoking	10	20.0	7	14.0	8	16.0	3	6.0
Diabetes mellitus	5	10.0	7	14.0	8	16.0	8	16.0
Coronary disease	2	4.0	2	4.0	1	2.0	1	2.0
Dyslipidemia	1	2.0	1	2.0	1	2.0	3	6.0
Family history								
Prostate cancer	7	14.0	5	10.0	10	20.0	6	12.0
Concomitant therapy*								
Any concomitant drug	32	64.0	34	68.0	38	76.0	33	66.0
ACEI	20	40.0	21	42.0	15	30.0	15	30.0
Diuretics	12	24.0	15	30.0	12	24.0	8	16.0
Calcium antagonists	6	12.0	5	10.0	15	30.0	9	18.0
Oral hypoglycemic drugs	3	6.0	6	12.0	9	18.0	8	16.0
Antiplatelet drugs	3	6.0	0	0.0	2	4.0	0	0.0
β-blockers	5	10.0	9	18.0	8	16.0	1	2.0
Vasodilators	2	4.0	3	6.0	1	2.0	0	0.0
Anti-gout	3	6.0	0	0.0	4	8.0	0	0.0
Analgesics, NSAID	5	10.0	1	2.0	4	8.0	3	6.0
Cholesterol-lowering drugs	0	0.0	1	2.0	4	8.0	3	6.0

All comparison were not significant.

Continuous variables (*t*-test for paired samples), categorical variables (*χ*^2^ test).

ACEI, angiotensin converting enzyme inhibitors; BPH, benign prostatic hyperplasia; IPSS, international prostate symptom score; LUTS, lower urinary tract symptoms; n, number of patients; NSAID, non-steroidal anti-inflammatory drugs; SD, standard deviation; *X*, mean.

*Consumed by ≥2 patients.

**Table 2.** Effects on Flowmetric Parameters

Treatment	Baseline	6 Months	Changes (%)
Maximum urinary flow (Qmax) (*X* ± SD) (mL/seg)			
Palmex	13.7 ± 9.8	17.5 ± 6.9^**^	+27.7
Saw palmetto	15.6 ± 7.6	19.2 ± 7.7^**^	+23.1
Finasteride	14.7 ± 9.6	18.9 ± 7.3^**^	+28.6
Tamsulosin	15.6 ± 9.1	19.7 ± 7.9^**^	+26.3
Mean flow (*X* ± SD) (mL/seg)			
Palmex	6.9 ± 4.4	8.8 ± 5.7^**^	+27.5
Saw palmetto	7.8 ± 3.6	9.6 ± 3.7^**^	+23.1
Finasteride	7.4 ± 4.4	9.4 ± 4.8^**^	+27.0
Tamsulosin	7.8 ± 3.8	9.8 ± 4.8^**^	+25.6
Voiding time (seg)			
Palmex	41.9 ± 19.1	35.8 ± 18.0^*^	−14.6
Saw palmetto	46.2 ± 25.1	37.0 ± 21.7^**^	−19.9
Finasteride	39.5 ± 24.8	30.2 ± 18.3^**^	−23.5
Tamsulosin	44.1 ± 22.1	35.0 ± 23.3^*^	−20.6

Comparison among groups were not significant (ANOVA).

SD, standard deviation; *X*, mean.

*P < .05, **P < .01 Comparison with baseline (*t*-test for paired samples).

**Table 3.** Effects on International Prostate Symptom Score (IPSS)

Treatment	Baseline (*X* ± SD)	2 Months (*X* ± SD)	Changes (%)	4 Months (*X* ± SD)	Changes (%)	6 Months (*X* ± SD)	Changes (%)
Palmex	10.8 ± 4.6	4.0 ± 2.5^*^	3**−58.5**	2.6 ± 1.7^*^	3**73.2**	2.3 ± 2.1^*^	3**74.2**
Saw palmetto	10.9 ± 4.5	4.5 ± 3.1^*^	3**−56.2**	3.2 ± 2.6^*^	3**67.8**	2.7 ± 2.5^*^	3**74.6**
Finasteride	10.0 ± 4.5	4.1 ± 2.8^*^	3**−49.9**	2.6 ± 1.9^*^	3**66.6**	2.5 ± 3.2^*^	3**60.3**
Tamsulosin	11.7 ± 4.5	4.0 ± 2.7^*^	3**−61.9**	3.0 ± 1.9^c^	3**70.7**	2.8 ± 2.1^*^	3**74.2**

Comparison among groups were not significant (ANOVA).

SD, standard deviation; *X*, mean.

*P < .001 Comparison with baseline (*t-*student for paired samples).

**Table 4.** Effects on Prostate Size and Residual Post-Voiding Volume

Treatment	Baseline	6 Months	Changes (%)
Prostate size (cm^3^) (*X* ± SD)			
Palmex	34.2 ± 19.7	28.1 ± 17.1^**^	3**−13.5 **
Saw palmetto	41.2 ± 35.3	34.5 ± 21.6^*^	3**−10.6 **
Finasteride	38.7 ± 25.8	29.1 ± 16.9^**^	3**−13.0 **
Tamsulosin	42.9 ± 27.1	38.6 ± 20.9	3**−10.0 **
Residual post-voiding volume (cm^3^) (*X* ± SD)			
Palmex	24.2 ± 26.7	14.3 ± 18.4^**^	3**−50.0 **
Saw palmetto	28.4 ± 38.8	20.4 ± 21.2	3**−41.2 **
Finasteride	18.2 ± 30.8	15.0 ± 18.6	3**−45.2**
Tamsulosin	21.1 ± 26.2	17.6 ± 20.8	3**−42.9 **

Comparison among groups were not significant (ANOVA).

SD, standard deviation; *X*, mean.

*P < .05, **P < .001 Comparison with baseline (*t*-test for paired samples).

**Table 5.** Adverse Events Referred During the Study

3**Adverse Events (AE)**	3**Palmex (n = 50)**		3**Saw palmetto (n = 50)**		3**Finasteride (n = 50)**		3**Tamsulosin (n = 50)**	
	3**n**	3**%**	3**n**	3**%**	3**n**	3**%**	3**n**	3**%**
Cystitis	0	0.0	1	2.0	1	2.0	0	0.0
Intestinal colic pain	0	0.0	1	2.0	0	0.0	0	0.0
Dizziness	0	0.0	0	0.0	1	2.0	0	0.0
Complete urinary retention	1	2.0	1	2.0	0	0.0	1	2.0
Decreased libido	0	0.0	1	2.0	0	0.0	1	2.0
Ejaculation disorders^+^	3**0** ^*^	3**0.0**	3**1** **^t^**	3**2.0**	3**0** ^*^	3**0.0**	3**6**	3**12.0**
Patients reporting AE	3**1**	3**2.0**	3**3**	3**6.0**	3**2**	3**4.0**	3**5**	3**10.0**
Patients with sexual related AE	3**0** ^**^	3**0.0**	3**2**	3**4.0**	3**0** ^**^	3**0.0**	3**7**	3**14.0**

Comparison among the groups was not significant (*χ*^2^ test).

n, number of patients.

^*^P < .05, ^**^P < .01, ^t^*P *= .056. ^+^Ejaculation disorders include retrograde and reduced volume ejaculation. Comparisons vs. Tamsulosin group. Fisher’s exact probability test.

## Data Availability

The data are part of all the documentation corresponding to the completed clinical study filed in the Quality Assurance Department of the Clinical Trials Unit of the National Center for Scientific Research and in the Department of Data Management and Statistical Processing of the National Coordinating Center for Clinical Trials of the Republic of Cuba, in which they will be kept for a period of 10 years, according to established standard work procedures.
